# Metabolic Phenotype Predicts Biochemical Response to Inositol Supplementation in Polycystic Ovary Syndrome: A Systematic Review and Meta‐Analysis

**DOI:** 10.1111/cen.70140

**Published:** 2026-04-07

**Authors:** Daniele Tienforti, Gennaro Puocci, Claudia Venditti, Valentina Gizzi, Carlo Pisanò, Elisa Cocci, Elisabetta Perfetto, Francesco D'Alessandro, Luca Spagnolo, Marco Giorgio Baroni, Arcangelo Barbonetti

**Affiliations:** ^1^ Andrology Unit, Department of Clinical Medicine, Life, Health and Environmental Sciences University of L'Aquila L'Aquila Italy

**Keywords:** D‐chiro‐Inositol, hyperandrogenism, inositol, insulin resistance, myo‐inositol, polycystic ovary syndrome, precision medicine

## Abstract

**Objective:**

To evaluate the effect of inositol supplementation on biochemical hyperandrogenism in women with polycystic ovary syndrome (PCOS) and to explore whether metabolic phenotype modifies the endocrine response.

**Design:**

Systematic review and meta‐analysis of randomized controlled trials conducted in accordance with PRISMA guidelines.

**Patients:**

Women diagnosed with PCOS according to established criteria (NIH, Rotterdam, or AE‐PCOS). Nine eligible trials comprising a total of 440 participants were included.

**Measurements:**

Primary outcomes were serum total testosterone (TT), calculated free testosterone (cFT), free androgen index (FAI), and sex hormone–binding globulin (SHBG). Prespecified subgroup analyses explored differences according to body mass index (BMI) and insulin resistance (HOMA‐IR).

**Results:**

Inositol supplementation was associated with significant reductions in TT (SMD –1.30; 95% CI –2.17 to –0.42), cFT, and FAI, together with an increase in SHBG. Substantial heterogeneity was observed for several outcomes. In subgroup analyses, the largest and most consistent reduction in TT was observed among normal‐weight women (BMI < 25 kg/m²) (SMD –2.97; 95% CI –3.78 to –2.16), with minimal heterogeneity (*I*² = 9%). No significant improvements were detected in overweight or obese women, nor in women with insulin resistance when considered independently of BMI. These subgroup findings should be interpreted as exploratory.

**Conclusions:**

Inositol supplementation is associated with improvements in biochemical hyperandrogenism in women with PCOS, with evidence of phenotype‐dependent variability. The most consistent biochemical response was observed in normal‐weight individuals. These findings support a metabolically informed, hypothesis‐generating framework and highlight the need for adequately powered, phenotype‐stratified trials incorporating clinically meaningful outcomes, including standardized measures of hirsutism, ovulatory function, and patient‐reported endpoints.

## 1 Introduction

Polycystic ovary syndrome (PCOS), first described by Stein and Leventhal in 1935 [1], remains one of the most prevalent endocrine disorders among reproductive‐age women. Hyperandrogenism is a core feature of the condition and represents a central component across all major diagnostic frameworks, including the National Institutes of Health/National Institute of Child Health and Human Development (NIH/NICHD) criteria [2], the Rotterdam European Society of Human Reproduction and Embryology/American Society for Reproductive Medicine (ESHRE/ASRM) consensus [3], and the Androgen Excess (AE)‐PCOS Society definition [4, 5]. Contemporary reviews consistently describe AE as a unifying biological hallmark underlying the heterogeneity of PCOS phenotypes [6].

From a mechanistic perspective, ovarian androgen overproduction is largely driven by increased luteinizing hormone (LH) stimulation of thecal cells, leading to enhanced synthesis of androstenedione (A) and testosterone [7]. This endocrine alteration contributes to the clinical manifestations of hyperandrogenism, such as hirsutism, acne, and androgenic alopecia, and is tightly intertwined with the metabolic disturbances characteristic of PCOS. Insulin resistance (IR) plays a pivotal amplifying role in this process, as compensatory hyperinsulinemia directly stimulates thecal steroidogenesis and suppresses hepatic sex hormone–binding globulin (SHBG) production, thereby increasing circulating bioavailable androgens [8]. Accumulating evidence supports a bidirectional and self‐reinforcing relationship between AE across the reproductive lifespan in women with PCOS [9].

At the population level, meta‐analytic evidence indicates that components of the metabolic syndrome, including elevated Homeostasis Model Assessment of IR (HOMA‐IR), dyslipidemia, and central adiposity, are associated with greater severity of biochemical hyperandrogenism and increased cardiometabolic burden [10]. Complementary analyses further suggest that insulin sensitivity, glucose–insulin homeostasis, and fat distribution are key determinants of phenotypic variability in PCOS, often exceeding the explanatory value of body mass index (BMI) alone [11]. These observations underscore the relevance of metabolic profiling for understanding inter‐individual differences in endocrine presentation and therapeutic response.

In this context, insulin‐sensitizing interventions have attracted increasing interest as potential strategies to mitigate both metabolic dysfunction and AE in PCOS. Among these, myo‐inositol (MI) and D‐chiro‐inositol (DCI), physiological inositol isomers involved in intracellular insulin signaling, have been widely investigated for their metabolic and reproductive effects. Despite their extensive clinical use, however, the magnitude and consistency of their impact on biochemical markers of hyperandrogenism remain debated, and inter‐study heterogeneity is substantial.

Moreover, relatively few quantitative syntheses have focused specifically on androgen‐related biochemical endpoints, and even fewer have examined whether metabolic phenotype modifies endocrine responsiveness to inositol supplementation. To address this gap, the present systematic review and meta‐analysis aimed to evaluate the effects of MI, DCI, and combined formulations on key biochemical markers of hyperandrogenism in women with PCOS, with a pre‐specified focus on metabolic stratification. By exploring phenotype‐specific patterns, this study seeks to generate clinically relevant hypotheses regarding patient subgroups that may derive greater biochemical benefit from inositol‐based interventions.

## 2 Materials and Methods

### 2.1 Study Design and Reporting Standards

This systematic review and meta‐analysis were conducted in accordance with the Cochrane Collaboration recommendations and followed the Preferred Reporting Items for Systematic Reviews and Meta‐Analyses (PRISMA 2020) statement and its protocol extension (PRISMA‐P) [12, 14]. Although the present work primarily focuses on randomized interventional studies, selected methodological principles from the Meta‐analysis of Observational Studies in Epidemiology (MOOSE) guidelines were considered where applicable, particularly with regard to transparency of reporting and handling of heterogeneity [13]. The study protocol was prospectively registered in the PROSPERO registry (CRD42024558844).

### 2.2 Search Strategy and Study Selection

A comprehensive literature search of PubMed, Scopus, Web of Science, and the Cochrane Library was performed to identify English‐language studies published up to March 1, 2025, that evaluated the effects of inositol supplementation on biochemical markers of hyperandrogenism in women with PCOS. Search terms combined keywords related to PCOS (‘polycystic ovary syndrome’, ‘PCOS’, ‘PCOD’), inositols (‘inositol’, ‘myo‐inositol’, ‘D‐chiro‐inositol’), and androgen‐related outcomes (‘testosterone’, ‘DHEAS’, ‘androstenedione’, ‘SHBG’, ‘free androgen index’, ‘FAI’), using Boolean operators ‘AND’ and ‘OR’. Reference lists of eligible articles were manually screened to identify additional relevant studies.

After duplicate removal, titles and abstracts were screened independently, followed by full‐text assessment of potentially eligible articles. Inclusion criteria were: (1) randomized or non‐randomized interventional studies with longitudinal follow‐up; (2) comparison of MI, DCI, or combined formulations with a metabolically neutral comparator (placebo or folic acid); (3) inclusion of women diagnosed with PCOS according to accepted criteria (NIH, Rotterdam, or AE‐PCOS); and (4) reporting of at least one biochemical androgen‐related outcome. Exclusion criteria included descriptive or cross‐sectional designs, studies not fulfilling diagnostic criteria for PCOS, interventions unrelated to androgen outcomes, and insufficient or unclear data. The study selection process is summarized in a PRISMA 2020 flow diagram [14].

### 2.3 Quality Assessment

Methodological quality was assessed using the Effective Public Health Practice Project (EPHPP) tool [15], a validated instrument applicable to both randomized and non‐randomized intervention studies [16]. The tool evaluates six domains (selection bias, study design, confounding, blinding, data collection methods, and withdrawals/drop‐outs), each rated as strong, moderate, or weak. Overall study quality was classified as strong (no weak domains), moderate (one weak domain), or weak (two or more weak domains). Quality assessment was incorporated into the interpretation of findings, particularly in relation to potential performance and detection bias for hormonal outcomes.

### 2.4 Data Extraction and Outcomes

For each included study, standardized mean differences (SMDs) between intervention and control groups were extracted or calculated for total testosterone (TT), androstenedione (A), dehydroepiandrosterone sulfate (DHEAS), 17‐hydroxyprogesterone (17OH‐P), SHBG, calculated free testosterone (cFT), and the FAI. When free testosterone was not directly reported, it was calculated using Vermeulen's equation via the ISSM calculator [17]. The FAI was calculated as TT (ng/dL) divided by SHBG (nmol/L), multiplied by 100.

Additional variables extracted included first author, publication year, country, study design, sample size, participant age, inositol formulation and dosage, comparator, treatment duration, and baseline metabolic parameters (BMI, fasting glucose, fasting insulin, and HOMA‐IR), when available.

### 2.5 Metabolic Stratification and Subgroup Definitions

Prespecified subgroup analyses were conducted according to BMI and IR status to explore potential sources of heterogeneity. BMI categories were defined using a threshold of 25 kg/m².

IR was primarily classified using the HOMA‐IR, with a cutoff > 4 to identify clinically relevant IR. This threshold was selected to capture marked metabolic impairment rather than borderline IR and is consistent with values commonly used in PCOS research. In support of this choice, population‐based data from the Study of Cardiovascular Risk in Adolescents (ERICA; 22,682 adolescent girls) reported 90th percentile HOMA‐IR values ranging from 3.6 to 4.3 across age and pubertal strata, suggesting that values around 4 correspond to the upper tail of the distribution and reflect substantial IR [18].

The authors acknowledge that HOMA‐IR cutoffs are not universally standardized and that methods for insulin and glucose measurement varied across included studies; these limitations were considered in the interpretation of subgroup findings. Sensitivity analyses using alternative HOMA‐IR thresholds or continuous modeling were not feasible due to incomplete reporting of metabolic data across trials.

### 2.6 Statistical Analysis

Pooled effect sizes were calculated using random‐effects models to account for between‐study variability, and are presented as SMDs with 95% confidence intervals. Statistical heterogeneity was assessed using Cochran's Q test and the *I*² statistic, with *I*² values ≥ 50% indicating substantial heterogeneity [19]. Given the expected clinical and methodological diversity across trials, heterogeneity was anticipated a priori, and subgroup analyses were conducted as exploratory and hypothesis‐generating.

Publication bias was assessed for outcomes reported in a sufficient number of studies using Doi plots and the Luis Furuya‐Kanamori (LFK) index, with values within ±1 indicating no asymmetry, ±1 to ±2 minor asymmetry, and >±2 major asymmetry [20]. Statistical analyses were performed using Review Manager (RevMan version 5.4) and R (metafor package, version 3.6.3).

## 3 Results

### 3.1 Study Selection and Quality Assessment

**Figure 1:**
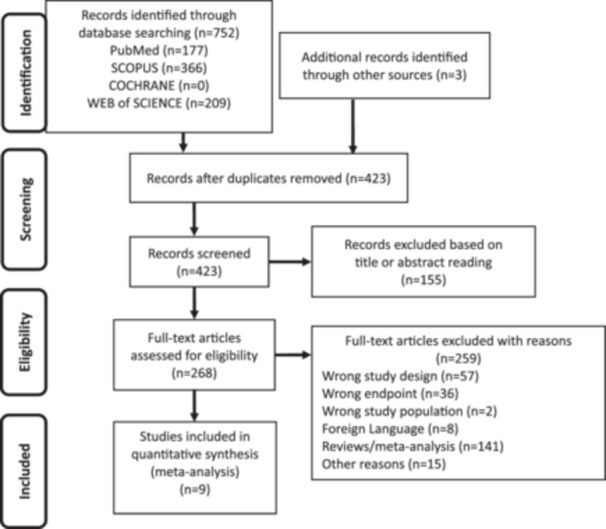
Flow chart showing an overview of the study selection process.

The electronic search yielded 752 records. After removal of duplicates, 423 articles remained, of which 155 were excluded during title and abstract screening. As illustrated in the PRISMA flow diagram (Figure 1), 268 full‐text articles were assessed for eligibility, and 9 randomized controlled trials (RCTs) met the inclusion criteria [21, 22, 23, 24, 25, 26, 27, 28, 29]. The main characteristics of the included trials are summarized in Table 1. When reported, baseline variables such as age, BMI, and HOMA‐IR appeared comparable between intervention and control groups, although metabolic data were incompletely reported across studies.

**Table 1:** Main characteristics of the studies included.

Authors	Country	*N* (Int./controls)	Int. arm (daily dose)	Control (daily dose)	Follow‐up (weeks)	Age (years)		BMI* (Kg/m²)		Insulin* (µU/mL)		Glycemia* (mg/dL)		HOMA index*	
						Int.	Controls	Int.	Controls	Int.	Controls	Int.	Controls	Int.	Controls
Artini et al. [27]	Italy	50 (25/25)	MI (2 g) + FA (200 µg)	FA (400 µg)	12	34.9 ± 2.1	36.2 ± 2.3	26.5 ± 6.1	26.3 ± 6.8	11.4 ± 2.2	11.4 ± 1.3	NA	NA	2.5 ± 0.6	2.5 ± 0.4
Benelli et al. [29]	Italy	46 (21/25)	MI (55 mg)+DCI (14 mg)	FA (200 µg)	24	23 ± 6.8	25 ± 7.3	32 ± 4.8	31 ± 4.6	20.19 ± 8.14	18 ± 8	85 ± 5.96	86.2 ± 9.1	3.38 ± 1.97	3.48 ± 2.02
Costantino et al. [21]	Italy	42 (23/19)	MI (4 g) + FA (400 µg)	FA (400 µg)	12–16	28.8 ± 1.5	27.1 ± 1.4	22.8 ± 0.3	22.5 ± 0.3	32.5 ± 4.1	30.8 ± 7.3	87.6 ± 3.5	84.9 ± 5.8	7.03 ± 0.04	6.46 ± 0.10
Donà et al. [24]	Italy	26 (18/8)	MI (1,2 g)	Placebo	12	23.5 ± 2.1	23.6 ± 1.4	21.6 ± 1.9	21.9 ± 0.6	7.49 ± 4.44	6.75 ± 1.67	86.22 ± 5.76	85.14 ± 6.84	1.60 ± 0.99	1.40 ± 0.40
Genazzani et al. [26]	Italy	20 (10/10)	MI (2 g) + FA (200 µg)	FA (200 µg)	12	NA	NA	29 ± 1.6	27.8 ± 2.1	12.4 ± 2.2	12.8 ± 1.3	NA	NA	2.8 ± 0.6	2.6 ± 0.4
Iuorno et al. [22]	Venezuela/USA	20 (10/10)	DCI (600 mg)	Placebo	6–8	28.2 ± 1.5	26.5 ± 1.4	22.4 ± 0.3	22.1 ± 0.3	30.5 ± 4.1	28.8 ± 7.3	86.5 ± 3.5	83.8 ± 5.8	6.51 ± 0.04	5.96 ± 0.10
Nestler et al. [25]	Venezuela/USA	44 (22/22)	DCI (1200 mg)	Placebo	6–8	29 ± 6	26 ± 5	31.3 ± 2.4	31.0 ± 2.2	35 ± 40	38 ± 51	86 ± 12	95 ± 21	7.43 ± 1.19	8.91 ± 2.64
Singh et al. [28]	India	132 (66/66)	MI (4 g)	FA (5 mg)	12	NA	NA	31.7 ± 1.64	31.97 ± 1.4	18.5 ± 1.93	18.42 ± 1.95	100.53 ± 9.9	101.11 ± 10.69	4.57 ± 0.05	4.59 ± 0.05
Yazdanpanah et al. [23]	Iran	60 (30/30)	MI (2 g) + FA (2 mg)	FA (1 mg)	6	27.77 ± 3.3	28.43 ± 3.07	27.32 ± 3.48	28.13 ± 2.99	19.67 ± 3.8	19.44 ± 3.28	NA	NA	NA	NA

*Note:* Data were expressed as mean ± standard deviation. *Data at the baseline.

Abbreviations: BMI, body mass index; CCT, controlled clinical trial; DCI, D‐chiro‐inositol; FA, folic acid; HOMA, homeostatic model assessment; Int., Intervention; MI, myo‐inositol; NA, not available.

### 3.2 Assessment of Study Quality

Methodological quality, assessed using the EPHPP tool, is presented in Supporting Information S1: Table 1. Three studies [22, 23, 25] were rated as strong, four [21, 24, 27, 28] as moderate, and one [26] as weak. The domains ‘study design’ and ‘data collection methods’ most frequently received strong ratings. In contrast, ‘blinding’ was the domain most often rated as weak, with only three studies explicitly reporting blinding of both participants and outcome assessors, a limitation particularly relevant for hormonal outcomes.

### 3.3 Study Characteristics

All nine included studies had a prospective interventional design and collectively enrolled 440 women with PCOS. Of these, 225 participants received inositol supplementation (172 MI, 32 DCI, and 21 combined MI + DCI), while 215 received a metabolically neutral comparator (placebo or folic acid). Intervention durations ranged from 6 to 24 weeks.

### 3.4 Overall Effects on Biochemical Hyperandrogenism

**Figure 2:**
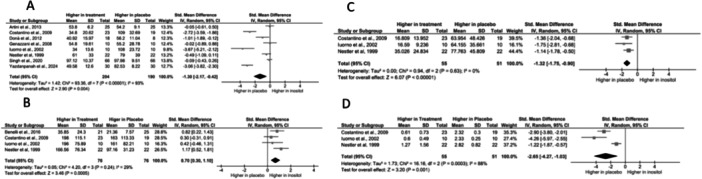
Forest plots showing the pooled effects of inositol supplementation on androgen‐related biomarkers in women with PCOS. Panels illustrate the standardized mean differences (SMDs) for: (A) total testosterone (TT), (B) calculated free testosterone (cFT), (C) free androgen index (FAI), and (D) sex hormone‐binding globulin (SHBG). Results derive from random‐effects models. Negative SMDs indicate higher levels in the placebo/control group (greater improvement with inositol), whereas positive SMDs indicate higher levels in the inositol group. Squares represent study‐specific effect sizes proportional to their weight, and diamonds indicate pooled estimates with corresponding 95% confidence intervals.

Across all included studies, inositol supplementation was associated with significant improvements in several biochemical markers of AE, although substantial between‐study heterogeneity was observed for some outcomes. TT was significantly reduced in the inositol group compared with controls (SMD –1.30; 95% CI –2.17 to –0.42; *p* = 0.004), with considerable heterogeneity (*I*² = 93%). cFT showed a consistent reduction (SMD –1.32; 95% CI –1.75 to –0.90; *p* < 0.00001) with no detectable heterogeneity (*I*² = 0%). Similarly, the FAI was significantly reduced (SMD –2.65; 95% CI –4.27 to –1.03; *p* = 0.001), although heterogeneity remained high (I² = 88%). SHBG levels increased significantly following inositol supplementation (SMD + 0.70; 95% CI 0.30 to 1.10; *p* = 0.0005), with low‐to‐moderate heterogeneity (*I*² = 29%) (Figure 2).

Among additional androgen‐related biomarkers, a significant reduction was observed for androstenedione (A) (SMD –0.64; 95% CI –1.08 to –0.19; *p* = 0.005; *I*² = 71%) and DHEAS (SMD –0.74; 95% CI –1.43 to –0.04; *p* = 0.04; *I*² = 75%) (Supporting Information S1: Figures 1A–B). In contrast, 17OH‐P showed a non‐significant trend toward increase (SMD + 0.44; 95% CI –0.03 to 0.92; *p* = 0.07; I*²* = 0%) (Supporting Information S1: Figure 1C).

### 3.5 Subgroup Analyses by BMI and IR

**Figure 3:**
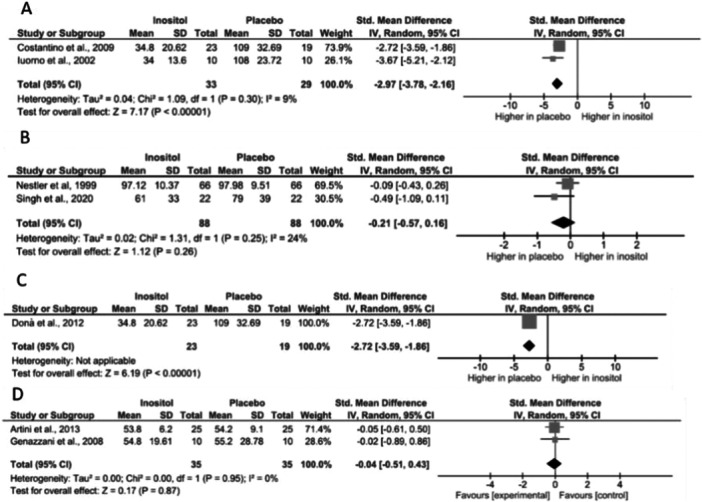
Forest plots showing the effects of inositol supplementation on individual androgen‐related outcomes in women with PCOS. Panels illustrate the standardized mean differences (SMDs) for: (A) total testosterone (TT) in normal‐weight women (BMI < 25 kg/m²), (B) TT in women with insulin resistance (HOMA‐IR > 4), (C) TT in normal‐weight women (BMI < 25 kg/m²) from single‐study analysis, and (D) androstenedione levels. Effect sizes were computed using random‐effects models. Negative SMDs indicate lower androgen levels in the inositol group compared with controls. Squares represent study‐specific estimates weighted by inverse variance, and diamonds denote the pooled effect with 95% confidence intervals.

Given the substantial heterogeneity observed in the primary analysis of TT, prespecified subgroup analyses were performed according to BMI and IR status (Figure 3).

In women with normal body weight (BMI < 25 kg/m²), TT was evaluated in two RCTs [21, 22], including a total of 62 participants (33 treated with inositols and 29 controls). In this subgroup, inositol supplementation was associated with a marked reduction in TT (SMD –2.97; 95% CI –3.78 to –2.16; *p* < 0.00001), with minimal between‐study heterogeneity (*I*² = 9%) (Figure 3A).

When analyses were restricted to women with IR (HOMA‐IR > 4), TT data were available from two studies [25, 28], comprising 176 participants (88 in the inositol group and 88 in the control group). In this subgroup, inositol supplementation was not associated with a statistically significant reduction in TT (SMD –0.21; 95% CI –0.57 to 0.16; *p* = 0.26), and heterogeneity was low (*I*² = 24%) (Figure 3B).

A single study [24] specifically evaluated normal‐weight women with preserved insulin sensitivity (BMI < 25 kg/m² and HOMA‐IR ≤ 4), including 42 participants (23 treated with inositols and 19 controls). In this isolated analysis, a large reduction in TT was observed (SMD –2.72; 95% CI –3.59 to –1.86; *p* < 0.00001) (Figure 3C). However, given the single‐study nature of this subgroup, these findings were considered exploratory and were not used to draw definitive conclusions.

Finally, androstenedione levels were assessed in two studies [26, 27], including 70 participants (35 in the inositol group and 35 controls). No significant effect of inositol supplementation on androstenedione was observed (SMD –0.04; 95% CI –0.51 to 0.43; *p* = 0.87), with no detectable heterogeneity (*I*² = 0%) (Figure 3D).

Overall, these subgroup analyses indicate that the pronounced reduction in TT associated with inositol supplementation was confined to normal‐weight women, whereas no significant effect was observed in insulin‐resistant women when considered independently of BMI. Given the limited number of studies contributing to each subgroup and the presence of single‐study analyses, these findings should be interpreted as hypothesis‐generating rather than confirmatory and primarily serve to inform future phenotype‐stratified trials.

### 3.6 Publication Bias

Publication bias was assessed for TT, the biochemical outcome most consistently reported across included trials (Supporting Information S1: Figure 3). The Doi plot showed mild asymmetry, with a LFK index of +1.08, indicating minor asymmetry and a low likelihood of clinically meaningful publication bias. The observed asymmetry is unlikely to materially affect the robustness of the pooled TT estimates.

## 4 Discussion

This meta‐analysis shows that inositol supplementation is associated with a clear improvement in biochemical hyperandrogenism in women with PCOS, with reductions in TT, cFT, and FAI, together with an increase in SHBG. However, these effects were not uniform across participants. The most consistent and homogeneous benefit was observed in normal‐weight women, whereas overweight or obese women and women with IR considered independently of BMI did not exhibit significant improvement.

This phenotype‐specific pattern aligns with established pathophysiological models in which IR amplifies ovarian androgen production and lowers SHBG through direct actions on thecal cells and hepatic metabolism [30]. Contemporary clinical reviews similarly highlight the bidirectional interplay between hyperinsulinemia and AE across PCOS phenotypes [7, 31]. At the same time, the absence of a significant effect in insulin‐resistant women when analyzed independently of BMI suggests that IR alone may be insufficient to predict endocrine responsiveness to inositol supplementation.

Our findings refine this framework by indicating that metabolic phenotype, rather than adiposity per se, may influence responsiveness to inositols. This interpretation is consistent with evidence showing that so‐called ‘lean PCOS’ often presents with visceral adiposity, impaired insulin signaling, and disproportionate cardiometabolic risk despite a normal BMI [32, 33]. The marked reduction in between‐study heterogeneity after stratification by BMI and HOMA‐IR further underscores the limited clinical utility of BMI alone and supports a metabolically informed approach to patient stratification.

The increase in SHBG observed with inositol supplementation provides additional indirect support for an improvement in insulin sensitivity. Although clinical signs of hyperandrogenism were inconsistently reported across included trials, the observed biochemical profile suggests a potential endocrine benefit. From a mechanistic standpoint, this effect is biologically plausible given the role of MI and DCI as intracellular insulin‐signaling second messengers and their documented capacity to improve endocrine and metabolic indices in women with PCOS [34].

Several limitations should be acknowledged. Treatment durations were relatively short, inositol formulations and dosages were heterogeneous, and clinical outcomes related to hyperandrogenism were incompletely reported, precluding quantitative synthesis. In addition, IR was primarily defined using HOMA‐IR, a surrogate marker with known variability across assays and populations and without universally accepted cutoffs; although the threshold adopted (> 4) was chosen to capture marked metabolic impairment and was supported by population‐based data, some degree of misclassification cannot be excluded [18]. Nevertheless, key strengths of this study include the focus on randomized trials, rigorous methodological quality appraisal, and pre‐specified metabolic stratification aimed at exploring sources of heterogeneity rather than post hoc subgrouping.

In conclusion, inositol supplementation improves biochemical hyperandrogenism in women with PCOS in a metabolically selective manner, with the most consistent effect observed in normal‐weight individuals. These findings support a phenotype‐oriented interpretation of inositol responsiveness and highlight the need for adequately powered, phenotype‐stratified randomized trials incorporating standardized clinical outcomes (including hirsutism scores, ovulatory function, and patient‐reported measures), in addition to biochemical endpoints, to determine whether endocrine improvements translate into tangible patient benefit.

## Author Contributions

Conceptualization: Daniele Tienforti, Arcangelo Barbonetti. Methodology: Daniele Tienforti, Gennaro Puocci, Arcangelo Barbonetti. Data Curation: Gennaro Puocci, Claudia Venditti, Valentina Gizzi, Elisabetta Perfetto, Elisa Cocci, Francesco D'Alessandro, Carlo Pisanò. Formal Analysis: Daniele Tienforti, Luca Spagnolo, Gennaro Puocci. Investigation: Gennaro Puocci, Claudia Venditti, Valentina Gizzi, Elisabetta Perfetto, Elisa Cocci, Francesco D'Alessandro, Carlo Pisanò. Writing − Original Draft: Daniele Tienforti, Gennaro Puocci. Writing − Review and Editing: Daniele Tienforti, Arcangelo Barbonetti, Marco Giorgio Baroni, Luca Spagnolo. Supervision: Arcangelo Barbonetti, Marco Giorgio Baroni.

## Funding

The authors have nothing to report.

## Conflicts of Interest

The authors declare no conflicts of interest.

## Supporting information


**Supplementary table 1:** Quality assessment of studies included through Effective Public Health Practice Project (EPHPP) tool. **Supplementary Figure 1A:** Forest plot showing the pooled effect of inositol supplementation on serum androstenedione levels in women with PCOS. **Supplementary Figure 1B:** Forest plot showing the pooled effect of inositol supplementation on serum DHEAS levels in women with PCOS. Standardized mean differences (SMDs) were calculated using a random‐effects model. **Supplementary Figure 1C:** Forest plot showing the effect of inositol supplementation on serum 17hydroxy‐progesterone (17‐OHP) levels in women with PCOS. **Supplementary Figure 2A:** Forest plot showing the effect of inositol supplementation on total testosterone (TT) levels in women with PCOS, stratified by body mass index (BMI). **Supplementary Figure 2B:** Forest plot showing the effect of inositol supplementation on total testosterone (TT) levels in women with PCOS, stratified by insulin resistance according to HOMA‐IR. **Supplementary Figure 3:** Doi plot assessing publication bias for the meta‐analysis of total testosterone (TT).The Doi plot displays effect size (standardized mean difference, SMD) against study precision (1/SE).

## Data Availability

All data included in this systematic review and meta‐analysis are derived from previously published studies. No new datasets were generated. Extracted data and analytic code are available from the corresponding author upon reasonable request.
