# Supplementary material for: Metabolic Phenotype Predicts Biochemical Response to Inositol Supplementation in Polycystic Ovary Syndrome: A Systematic Review and Meta‐Analysis
Source: Clin Endocrinol (Oxf). 2026 Apr 7;105(2):237–44. doi: 10.1111/cen.70140 (PMC13326987; doi:10.1111/cen.70140)
Supplement: Supplementary file 1 — Supplementary table 1: Quality assessment of studies included through Effective Public Health Practice Project (EPHPP) tool. Supplementary Figure 1A: Forest plot showing the pooled effect of inositol supplementation on serum androstenedione levels in women with PCOS. Supplementary Figure 1B: Forest plot showing the pooled effect of inositol supplementation on serum DHEAS levels in women with PCOS. Standardized mean differences (SMDs) were calculated using a random‐effects model. Supplementary Figure 1C: Forest plot showing the effect of inositol supplementation on serum 17hydroxy‐progesterone (17‐OHP) levels in women with PCOS. Supplementary Figure 2A: Forest plot showing the effect of inositol supplementation on total testosterone (TT) levels in women with PCOS, stratified by body mass index (BMI). Supplementary Figure 2B: Forest plot showing the effect of inositol supplementation on total testosterone (TT) levels in women with PCOS, stratified by insulin resistance according to HOMA‐IR. Supplementary Figure 3: Doi plot assessing publication bias for the meta‐analysis of total testosterone (TT).The Doi plot displays effect size (standardized mean difference, SMD) against study precision (1/SE). [file CEN-105-237-s001.docx]

**SUPPLEMENTARY MATHERIALS**

**Supplementary table 1.** Quality assessment of studies included through Effective Public Health Practice Project (EPHPP) tool.

|  | **Selection bias** | **Study design** | **Confounders** | **Blinding** | **Data collection methods** | **Withdrawals and Drop-outs** | **Global rating** |
| --- | --- | --- | --- | --- | --- | --- | --- |
| *Artini et al* | Moderate | Strong | Strong | Weak | Strong | Strong | Moderate |
| *Benelli et al.* | Strong | Strong | Strong | Weak | Strong | Strong | Moderate |
| *Costantino et al.* | Moderate | Strong | Strong | Strong | Strong | Strong | Strong |
| *Donà et al.* | Moderate | Strong | Moderate | Weak | Strong | Strong | Moderate |
| *Genazzani et al.* | Strong | Strong | Moderate | Weak | Moderate | Strong | Moderate |
| *Iuorno et al.* | Moderate | Strong | Strong | Strong | Strong | Strong | Strong |
| *Nestler et al.* | Strong | Strong | Moderate | Strong | Strong | Strong | Strong |
| *Singh et al.* | Moderate | Strong | Strong | Moderate | Moderate | Strong | Strong |
| *Yazdanpanah et al.* | Moderate | Strong | Moderate | Strong | Strong | Moderate | Moderate |

**Supplementary Figure 1A.** Forest plot showing the pooled effect of inositol supplementation on serum androstenedione levels in women with PCOS. Standardized mean differences (SMDs) were calculated using a random-effects model. Negative SMD values indicate lower androstenedione concentrations in the inositol group compared with controls. Squares represent individual study estimates weighted by their inverse variance, and the diamond denotes the pooled effect with its 95% confidence interval. Between-study heterogeneity was moderate (I² = 71%).


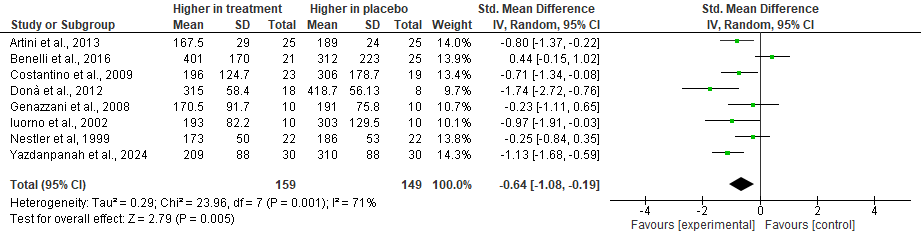


**Supplementary Figure 1B. Forest plot showing the pooled effect of inositol supplementation on serum DHEAS levels in women with PCOS.** Standardized mean differences (SMDs) were calculated using a random-effects model. Negative SMD values indicate lower DHEAS concentrations in the inositol group compared with controls. Individual study estimates are shown as squares proportional to study weight, and the diamond represents the pooled effect with its 95% confidence interval. Between-study heterogeneity was moderate (I² = 75%).


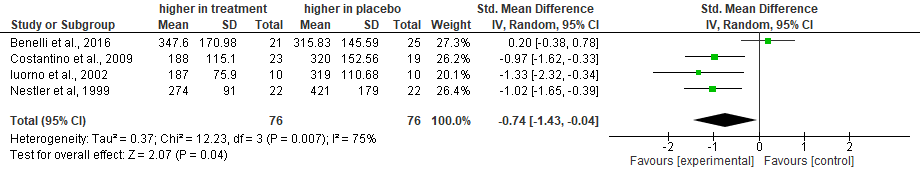


**Supplementary Figure 1C. Forest plot showing the effect of inositol supplementation on serum 17hydroxy-progesterone (17-OHP) levels in women with PCOS.** Standardized mean differences (SMDs) were estimated using a random-effects model. Positive SMD values indicate higher 17-OHP concentrations in the inositol group relative to controls. Squares indicate individual study estimates weighted by inverse variance, and the diamond represents the pooled effect with its 95% confidence interval. No between-study heterogeneity was detected (I² = 0%).


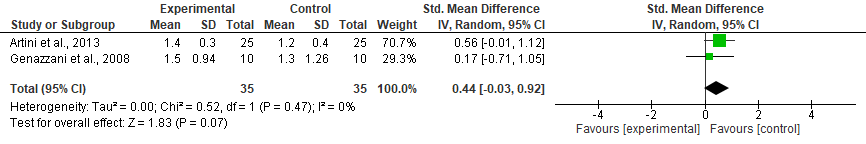


**Supplementary Figure 2A. Forest plot showing the effect of inositol supplementation on total testosterone (TT) levels in women with PCOS, stratified by body mass index (BMI).** Analyses were performed separately for women with BMI 21–25 kg/m² and BMI > 25 kg/m². Standardized mean differences (SMDs) were calculated using a random-effects model. Negative SMD values indicate lower TT concentrations in the inositol group compared with controls. Squares represent study-specific effect estimates weighted by inverse variance, and diamonds indicate pooled effects with 95% confidence intervals. Significant reductions in TT were observed only in the BMI 21–25 kg/m² subgroup, while no significant effect was detected in women with BMI > 25 kg/m². Substantial heterogeneity was present across studies.


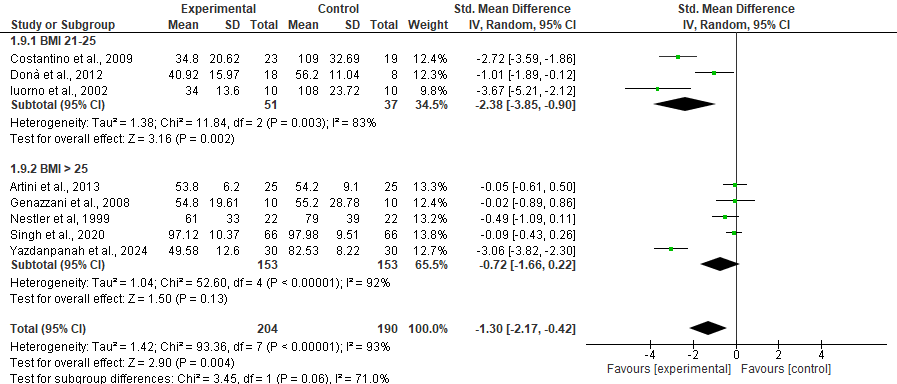


**Supplementary Figure 2B. Forest plot showing the effect of inositol supplementation on total testosterone (TT) levels in women with PCOS, stratified by insulin resistance according to HOMA-IR.** Subgroups include women with HOMA-IR > 4 and those with HOMA-IR < 4. Standardized mean differences (SMDs) were calculated using a random-effects model. Negative SMD values indicate lower TT concentrations in the inositol group compared with controls. Squares represent study-specific estimates weighted by inverse variance, and diamonds indicate pooled effects with 95% confidence intervals. A significant reduction in TT was observed in the HOMA-IR > 4 subgroup, while no significant effect was detected in women with HOMA-IR < 4. Between-study heterogeneity was substantial.


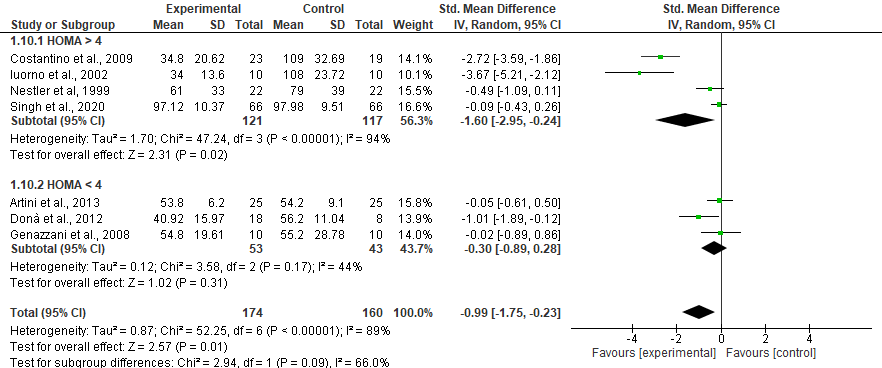


**Supplementary Figure 3.** Doi plot assessing publication bias for the meta-analysis of total testosterone (TT).
The Doi plot displays effect size (standardized mean difference, SMD) against study precision (1/SE). The Luis Furuya-Kanamori (LFK) index was +1.08, indicating minor asymmetry consistent with a low likelihood of publication bias.


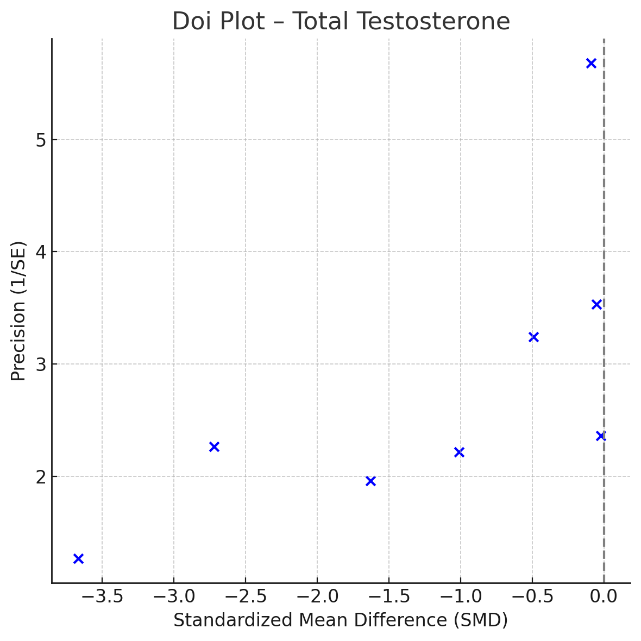


LFK index: +1.08
